# Surgical and anesthetic rewiring of the glymphatic system in postoperative cognitive dysfunction

**DOI:** 10.4103/NRR.NRR-D-25-01120

**Published:** 2026-01-02

**Authors:** Bandy Chen

**Affiliations:** Department of Medicine, UC San Diego School of Medicine, La Jolla, CA, USA

Postoperative cognitive dysfunction (POCD) is a condition characterized by a variety of neurological deficits, including memory deficits, difficulty with attention and executive function, and behavioral changes or delirium-like symptoms. These changes can be transient or long-lasting; however the exact mechanisms involved in POCD pathogenesis remain unclear. Well-established pathways that can contribute to POCD are neuroinflammation, blood-brain barrier disruption, and neurovascular dysfunction. An emerging candidate is the glymphatic system due to anesthesia’s profound effects on modulating glymphatic flow. This system is intimately linked to other brain processes, collectively referred to as the neuro-glial-vascular landscape (Chen et al., 2025a). The central hypothesis linking POCD and the glymphatic system is that surgical stress and anesthesia impair glymphatic function, which results in the accumulation of neurotoxic waste products (e.g., amyloid-beta and tau) in the brain. While this accumulation may not lead to symptoms in every individual, people with baseline glymphatic dysfunction, such as older individuals or individuals with neurological disorders, may be more susceptible to POCD. This perspective delves into the surgical and anesthetic impairment of glymphatic function, how the disruption can lead to POCD, and possible therapeutic interventions to mitigate these effects.

The combination of anesthesia and surgery has been shown to alter central homeostasis, particularly in the hippocampus, by inducing neuroinflammation and neurovascular uncoupling. In mice, peripheral surgery from osteotomy induces a dynamic response in the hippocampus characterized by metabolic, structural, and functional changes in astrocytes (Femenia et al., 2018). At 6 hours post-surgery, there is an elevation of proinflammatory cytokines in the hippocampus, consistent with previous findings indicative of an initial inflammatory response (Femenia et al., 2018). Interestingly, there is a reduction in aquaorin-4 (AQP4) and glial fibrillary acidic protein gene expression with a decrease in glial fibrillary acidic protein immunoreactivity at 24 hours post-surgery. This parallels other findings that report surgery-induced blood–brain barrier disruption at 24 hours. When extended to 72 hours, there is an increase in neuronal glutamatergic transmission with changes in AMPA (α-amino-3-hydroxy-5-methyl-4-isoxazolepropionic acid) receptor subunit, hinting at a disrupted glutamate-gamma-aminobutyric acid homeostasis (Femenia et al., 2018). Administration of anesthesia and surgery-derived extracellular vesicles induces delirium-like behavioral changes and hippocampal inflammation in control mice (Gao et al., 2025). Analysis of the extracellular vesicles indicate a marked contrast in the protein and miRNA profiles between surgery/anesthesia mice and controls (Gao et al., 2025). The protein SAA1 and miRNAs miR-103-3p and miR-31-5p are identified as potential mediators of POCD. *In vitro* overexpression of miR-103-3p in microglial cells reduces the viability and promotes the secretion of proinflammatory factors. Overall, these findings indicate a disruption in neuroglial metabolic coupling as a possible driver of POCD.

**Glymphatic dysfunction drives postoperative cognitive decline:** The discovery of the glymphatic system as the primary waste clearance apparatus in the brain has emerged as a potential mechanistic bridge between perioperative physiological stress and cognitive outcomes. Cerebrospinal fluid (CSF) dynamics, as a proxy for glymphatic activity, has been shown to be reduced following anesthesia and surgery. In humans, sevoflurane monoanesthesia for 2 hours decreases cisternal CSF flow and overall brain connectivity measured by blood oxygen signals in functional magnetic resonance imaging scans (Zimmermann et al., 2025). This impairment in global gray matter-CSF coupling persists up to 45 minutes after re-emergence from anesthesia, suggesting a delayed recovery of the glymphatic system (Zimmermann et al., 2025). Whether lingering neural activity changes after anesthesia alter CSF flow or are a consequence of glymphatic dysfunction remains to be elucidated, but it is evident that altered coupling between the two signals may partially explain POCD. The impact of these alterations likely varies across individuals; however, they may have clinical relevance in older adults or individuals with neurodegenerative disease, who often exhibit excitatory-inhibitory imbalance. In patients with obstructive sleep apnea, there is reduced glymphatic function 48 hours post-surgery with a decrease in general cognition scores and other specific memory domains (Roy et al., 2025). While the patients did not develop delirium, alterations in specific memory domains hint at a connection between glymphatic dysfunction and brain fogginess shortly after surgery (Roy et al., 2025). While these results are promising, CSF flow is an indirect measure of glymphatic function with inherent limitations. Future studies using the current gold standard, invasive intrathecal gadolinium, for assessing glymphatic flow are needed to draw more definitive conclusions.

In mice, exposure to isoflurane for 6 hours disrupts brain-wide glymphatic influx and efflux measured 3 days after anesthesia (Dong et al., 2024). The reduced glymphatic activity parallels elevated AQP4 depolarization and inflammatory protein accumulation in the hippocampus (Dong et al., 2024). These mice demonstrate reduced cognitive performance in both the fear condition test for associated learning and memory and Y maze test for working memory. Compared to adult mice (6 months), aged mice (18 months) exhibit delayed tracer influx 24 hours post-surgery (Chen et al., 2025c). This coincides with poorer T-maze performance, reinforcing that the aging brain is more sensitive to glymphatic dysfunction and cognitive decline (Chen et al., 2025c).

One hypothesis is that surgical trauma can aggravate POCD through preexisting cerebral lymphatic drainage impairment. In middle-aged mice with deep cervical lymph nodes ligation, performing a laparotomy 4 weeks after the initial surgery exacerbates glymphatic dysfunction and AQP4 depolarization with elevated A1 astrocytes and microglia activation and proinflammatory cytokine expression in the hippocampus (Zhu et al., 2024b). This is accompanied by neuronal loss and impairment in exploratory behavior and spatial working memory (Zhu et al., 2024b). Interestingly, the high mobility group box 1/Toll-like receptor 4/nuclear factor κB pathway is augmented after the laparotomy, hinting at a possible pathway that can be targeted for glymphatic recovery.

These findings highlight an age-dependent, neuroinflammation-driven glymphatic dysfunction that increases the risk of POCD. This suggests the potential to modulate the glymphatic system as a preventive and therapeutic target to mitigate POCD.

**Enhancing glymphatic flow for cognitive recovery:** Given the critical role of the glymphatic system in neurological disorders and cognitive function, modulation of glymphatic activity has been a promising target aimed at improving neurological function. While much of the attention has been towards neurodegenerative diseases such as Alzheimer’s disease and Parkinson’s disease, preliminary analyses that demonstrate therapeutic modulation of glymphatic flow show promising results in attenuating POCD. However, most of the evidence stem from preclinical models; therefore, the translational relevance to humans requires further investigation.

In mice, adeno-associated viruses-mediated overexpression of AQP4 enhances brain-wide glymphatic clearance and ameliorates cognitive impairments 3 days after isoflurane exposure (Dong et al., 2024). In contrast, postoperative pharmacological inhibition of AQP4 polarization using TGN-020 at 24 hours exacerbates neuroinflammation and cognitive deficits (Dong et al., 2024). Pretreatment with trifluoperazine, a calmodulin antagonist, alleviates postoperative anxiety-like behaviors and cognitive deficits by restoring AQP4 polarization and augmenting perivascular CSF tracer influx (Chen et al., 2025b). Pretreatment for 5 days and continued administration for 2 days following laparotomy with the matrix metalloproteinase-9 inhibitor GM6001 ameliorates cognitive deficits and neuroinflammation and preserves blood–brain barrier integrity and AQP4 localization in the hippocampus of aged mice (Zhu et al., 2024a). The proposed mechanism of AQP4 stabilization is through the inhibition of matrix metalloproteinase-9-mediated cleavage of dystroglycan, a key membrane anchor protein. These results suggest that enhancing AQP4 polarization is a key driver of glymphatic activity to ameliorate POCD.

Given the close association between sleep and glymphatic activity, therapeutics that can mimic or promote natural sleep patterns might improve glymphatic activity. This is crucial because while general anesthesia induces a loss of consciousness, it does not replicate natural sleep patterns and may disrupt normal sleep architecture and circadian rhythms. Young mice (P8) with multiple sevoflurane exposures demonstrate glymphatic dysfunction and AQP4 depolarization in the hippocampus (Wang et al., 2024). These mice exhibit long-term (P32) behavioral abnormalities with reduced performance in the Y-maze and novel object recognition tests (Wang et al., 2024). Cotreatment with dexmedetomidine ameliorates sevoflurane-induced alterations through the activation of the PI3K/AKT pathway (Wang et al., 2024). This study is consistent with another study that assesses glymphatic activity under different anesthesia regiments (Hablitz et al., 2019). One explanation is that dexmedetomidine acts as a selective α2-adrenergic receptor agonist, which reduces norepinephrine release and mimics natural non-REM sleep patterns. In contrast, sevoflurane induces unconsciousness through widespread neuronal suppression with incomplete norepinephrine suppression and disrupts natural sleep-like neural oscillations. These findings highlight the importance of choosing the right anesthetic when studying glymphatic function in preclinical models.

Melatonin is a hormone produced by the pineal gland that regulates sleep-wake cycles. Exogenous melatonin is primarily used as a sleep aid and has been found to enhance glymphatic flow. In aged mice, laparotomy under sevoflurane exerts a greater effect than sevoflurane alone, resulting in sleep disturbances, altered sleep-wake rhythm, and delirium-like behavior (Jia et al., 2024). These mice exhibit a peak shift in circadian rhythm gene expression, such as Bmal1, Clock, and Cry1, indicating a misalignment in the molecular circadian clock (Jia et al., 2024). This is accompanied by an increased expression of the MT1 melatonin receptor in the suprachiasmatic nucleus, suggesting a compensatory response to circadian disruption (Jia et al., 2024). There is reduced phosphorylation of extracellular signal-regulated kinase and cAMP response element binding protein in the hippocampus and prefrontal cortex, pointing to a suppression of memory consolidation and synaptic plasticity (Jia et al., 2024). Interestingly, treatment with melatonin ameliorates the behavioral deficits by restoring circadian rhythm and reversing melatonin receptor expression and extracellular signal-regulated kinase/cAMP response element binding protein signaling (Jia et al., 2024). One proposed mechanism underlying the efficacy of melatonin in POCD is its ability to regulate glymphatic flow. In a chronic stress mouse model, melatonin treatment alleviates depressive-like behaviors and cognitive deficits by restoring glymphatic function and normalizing AQP4 polarization and circadian protein rhythms (Yao et al., 2023). The exact mechanism by which melatonin enhances glymphatic function remains unclear. Due to sleep being a main driver of glymphatic function, it is likely that the ability of melatonin to improve sleep architecture provides an indirect mechanism to modulate glymphatic flow. Whether melatonin has a direct effect on AQP4 polarization is unclear.

Collectively, improving glymphatic flow by modulating natural sleep patterns is a promising way to minimize POCD. Both dexmedetomidine and melatonin are compelling prospects to augment glymphatic activity (**Figure 1**). While preclinical data on these agents are encouraging, their efficacy in preventing POCD in humans remains unproven. Future clinical trials that evaluate the optimal timing and dosing of these agents will be critical for reducing the incidence of POCD.

**Figure 1:**
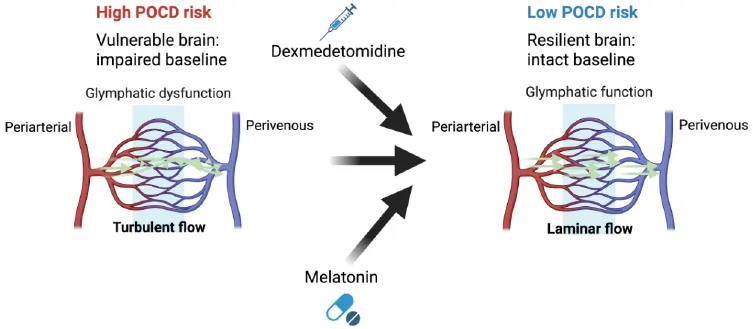
Perioperative disruption of glymphatic clearance drives cognitive decline. Anesthesia and surgery induce a neuroinflammatory landscape with glymphatic dysfunction. The lack of brain clearance results in the accumulation of neurotoxic molecules that can lead to neural imbalance (e.g., excitatory-inhibitory imbalance) and cognitive decline. To mitigate these effects, maintaining proper glymphatic activity throughout the perioperative period is essential. For example, the combination of preoperative melatonin and perioperative dexmedetomidine can enhance natural sleep patterns to promote glymphatic clearance. These interventions can be implemented either prophylactically (before or during surgery) or postoperatively. Created with BioRender.com. POCD: Postoperative cognitive dysfunction.

**Conclusions:** Surgical and anesthetic rewiring of the glymphatic system is a pathological feature that increases the risk for POCD. While the exact mechanisms on how glymphatic dysfunction occurs remain to be elucidated, it is evident that impaired CSF dynamics can lead to cognitive decline. While most preclinical evidence focused on the hippocampus, it will be fundamental to include other brain regions to offer a holistic view. This will reveal sites of vulnerabilities in response to perioperative factors. As an over-the-counter supplement, melatonin has the potential to minimize the risk of POCD and can be taken preoperatively. Different anesthetics can have varying effects on glymphatic activity; therefore, choosing the proper anesthetic for a given patient population is essential for optimizing outcomes. The combination of pre- and perioperative interventions may help mitigate both the frequency and severity of POCD.

## References

[R1] Chen B, Lenck S, Thomas JL, Schneeberger M (2025). The glymphatic system as a nexus between obesity and neurological diseases. Nat Rev Endocrinol.

[R2] Chen C, Zhu B, Luo W, Cao A, Zhou W, Weng Y, Wang J (2025). Trifluoperazine improves postoperative cognition by influencing astrocyte endfoot morphology and aquaporin-4 polarity. Mol Neurobiol.

[R3] Chen K, Du X, Chao MA, Xie Z, Yang G (2025). Surgery impairs glymphatic activity and cognitive function in aged mice. Mol Brain.

[R4] Dong R, Han Y, Lv P, Jiang L, Wang Z, Peng L, Liu S, Ma Z, Xia T, Zhang B, Gu X (2024). Long-term isoflurane anesthesia induces cognitive deficits via AQP4 depolarization mediated blunted glymphatic inflammatory proteins clearance. J Cereb Blood Flow Metab.

[R5] Femenia T, Gimenez-Cassina A, Codeluppi S, Fernandez-Zafra T, Katsu-Jimenez Y, Terrando N, Eriksson LI, Gomez-Galan M (2018). Disrupted neuroglial metabolic coupling after peripheral surgery. J Neurosci.

[R6] Gao Y, Tang S, Liu J, Yang X, Chen H, Li J, Ni X (2025). The administration of circulating extracellular vesicles modified by anesthesia and surgery induces delirium-like behaviors in aged mice. CNS Neurosci Ther.

[R7] Hablitz LM, Vinitsky HS, Sun Q, Stæger FF, Sigurdsson B, Mortensen KN, Lilius TO, Nedergaard M (2019). Increased glymphatic influx is correlated with high EEG delta power and low heart rate in mice under anesthesia. Sci Adv.

[R8] Jia X, Song Y, Li Z, Yang N, Liu T, Han D, Sun Z, Shi C, Zhou Y, Shi J, Liu Y, Guo X (2024). Melatonin regulates the circadian rhythm to ameliorate postoperative sleep disorder and neurobehavioral abnormalities in aged mice. CNS Neurosci Ther.

[R9] Roy B, Kumar R, Sarovich SD, Vacas S (2025). The role of the glymphatic system in perioperative neurocognitive disorders. J Neurosurg Anesthesiol.

[R10] Wang S, Yu X, Cheng L, Ren W, Wen G, Wu X, Lou H, Ren X, Lu L, Hermenean A, Yao J, Li B, Lu Y, Wu X (2024). Dexmedetomidine improves the circulatory dysfunction of the glymphatic system induced by sevoflurane through the PI3K/AKT/ΔFosB/AQP4 pathway in young mice. Cell Death Dis.

[R11] Yao D, Li R, Hao J, Huang H, Wang X, Ran L, Fang Y, He Y, Wang W, Liu X, Wang M (2023). Melatonin alleviates depression-like behaviors and cognitive dysfunction in mice by regulating the circadian rhythm of AQP4 polarization. Transl Psychiatry.

[R12] Zhu B, Cao A, Chen C, Zhou W, Luo W, Gui Y, Wang Q, Xu Z, Wang J (2024). MMP-9 inhibition alleviates postoperative cognitive dysfunction by improving glymphatic function via regulating AQP4 polarity. Int Immunopharmacol.

[R13] Zhu X, Lin J, Yang P, Wu S, Lin H, He W, Lin D, Cao M (2024). Surgery induces neurocognitive disorder via neuroinflammation and glymphatic dysfunction in middle-aged mice with brain lymphatic drainage impairment. Front Neurosci.

[R14] Zimmermann J, Sorg C, Muller L, Zistler F, Neumaier V, Bonhoeffer M, Ranft A, Golkowski D, Priller J, Zimmer C, Ilg R, Preibisch C, Schneider G, Nuttall R, Zott B (2025). Impaired macroscopic cerebrospinal fluid flow by sevoflurane in humans during and after anesthesia. Anesthesiology.

